# The seeded growth of graphene

**DOI:** 10.1038/srep05682

**Published:** 2014-07-14

**Authors:** Jae-Kap Lee, Sohyung Lee, Yong-Il Kim, Jin-Gyu Kim, Bong-Ki Min, Kyung-Il Lee, Yeseul Park, Phillip John

**Affiliations:** 1 Interface Control Research Center, Korea Institute of Science and Technology (KIST), Seoul 130-650, Korea; 2 Department of Semiconductor Science, Dongguk University, Seoul 100-715, Korea; 3 Korea Research Institute of Standards and Science, Daejeon 305-600, Korea; 4 Division of Electron Microscopic Research, Korea Basic Science Institute, Daejeon 305-333, Korea; 5 Center for Spintronics Research, KIST, Seoul 130-650, Korea; 6 Instrumental Analysis Center, Yeungnam University, Daegu 712-749, Korea; 7 School of Engineering and Physical Sciences, Heriot-Watt University, Edinburgh EH14 4AS, UK

## Abstract

In this paper, we demonstrate the seeded growth of graphene under a plasma chemical vapor deposition condition. First, we fabricate graphene nanopowders (~5 nm) by ball-milling commercial multi-wall carbon nanotubes. The graphene nanoparticles were subsequently subject to a direct current plasma generated in a 100 Torr 10%CH_4_ - 90%H_2_ gas mixture. The plasma growth enlarged, over one hour, the nuclei to graphene sheets larger than one hundred nm^2^ in area. Characterization by electron and X-ray diffraction, high-resolution transmission electron microscopy images provide evidence for the presence of monolayer graphene sheets.

Graphene layers, as a result of weak van der Waals attraction, cause the material to adopt a stacked multilayer configuration^1^ and make it difficult to deposit or fabricate a perfect 2-dimensional structure^2^,^3^. For example, graphene formed on a metal substrate by chemical vapour deposition (CVD) appears as single and multiple graphene layers^4^,^5^,^6^ which may be regarded as azimuthally aligned graphite rather than graphene. Furthermore, monolayer graphene (~0.4 nm in thickness) is very seldom produced even in the samples prepared by micromechanical cleavage^2^,^7^,^8^. The yield of graphene via a mass production chemical route^9^,^10^,^11^,^12^,^13^ may be also very low because the residues in the centrifuged supernantant liquid are only ~0.5% of the starting material^9^. The chemical approach to fabricating monolayer graphene has two fundamental problems. First, it is energetically difficult for molecules to intercalate the graphite layers. For example, intercalation of relatively small lithium atoms into graphite requires >0.5 eV/atom^14^. Secondly, isolated graphene planes may restack during further treatment in suspension. The relative effective area of graphene for van der Waals interactions is much larger, by approximately two orders of magnitude, than that of untreated single-wall carbon nanotubes (SWNTs) which exist as bundles^15^. Indeed, there have been no reports showing direct evidence of the presence of monolayer graphene powders, such as broadened 002 peaks in X-ray diffraction (XRD) patterns together with high-resolution transmission electron microscopy (HRTEM) images showing separated monolayer graphene although there are many reports^9^,^10^,^11^,^12^,^13^,^16^,^17^ of the large-scale production of graphene.

Recently we have shown that multi-wall carbon nanotubes (MWNTs) are composed of AA′ stacked graphene helices where width is of the order of a few nanometers^18^, unlike the conventional view of the structure of MWNTs: a tubule comprising concentrically nested graphene sheets^19^. This observation suggests that shortening helical graphitic materials, exhibiting a high aspect ratio (~1,000), may form nano-scale monolayer graphene. In this work, we fabricate graphene nanopowders (GNPs) with a size of ~5 nm by ball-milling commercial MWNTs, and demonstrate that the GNPs used as nuclei grow to monolayer graphene sheets under a plasma CVD condition.

## Results

**Figure 1:**
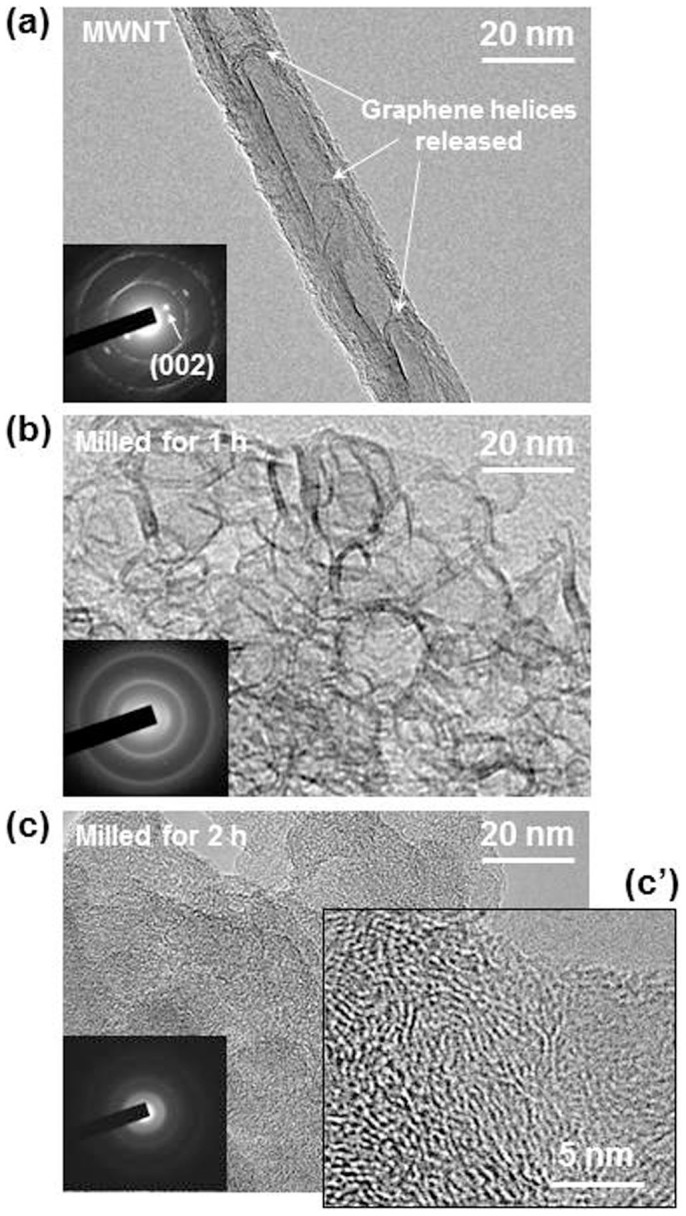
HRTEM images showing a mechanical conversion of MWNTs into GNPs. (a), Pristine VCCVD-MWNT showing the graphene helices released from the walls. (b), Milled for 1 hour. (c), (c′), Milled for 2 hours.

Figure 1 shows HRTEM images before (a) and after (b and c) mechanical milling of MWNTs for varying periods of time. The pristine tubules reveal the unique bamboo morphology^18^,^19^ evident of our helical MWNTs^18^. With an hour of milling, the tubules were decomposed to graphitic nanoribbons whose thickness and width are ~5 nm, respectively (b). The graphitic nanoribbons were cleaved into graphene nanopowders (GNPs) of a few nm in length with another 1 hour milling (c), as shown in Fig. 1c′. GNPs are randomly oriented although stacked graphene fringes are partially observed. The variation of electron diffraction (ED) patterns (inset) is consistent with the dramatic change of the materials from tubular MWNTs (a) where the 002 spots appear as two spots, via stacked nanoribbons (b), to GNPs (c) where the 002 spots are transformed into a diffused ring. The ED pattern of the sample milled for 2 hours exhibited diffuse rings (Fig. 1c) providing evidence for the presence of graphene in the form of powders.

**Figure 2:**
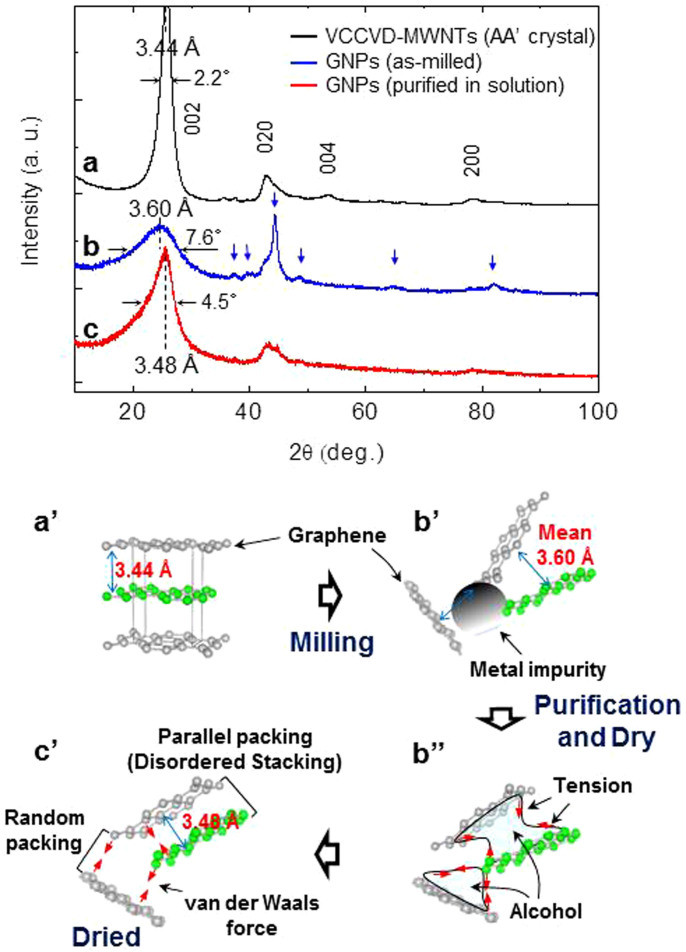
XRD patterns of the samples and schematic explaining the states of the samples with the processes. (a), XRD pattern of pristine VCCVD-MWNTs where characteristic peaks are assigned to an orthorhombic AA′ crystal. (b), XRD pattern of as milled GNPs where 002 peak is broadened with milling for 2 hours. Blue arrows indicate signals of metal impurities originated from the steel balls. (c), XRD pattern of purified GNPs where 002 peak is evolved after purification. Ordered graphene layers (a′) are scattered by milling and exist disorderly with metal impurities (b′). During dry after purification graphene layers are packed by tension of alcohol (b″). The packing can be classified as either parallel or random packing (c′).

XRD analysis of the samples provides more specific information as to the structural changes of the MWNTs following milling. The data showed that 002, 020, 004, and 200 peaks of orthorhombic AA′ graphene stacked MWNTs^16^ broadened with 2 hours milling, as shown in Fig. 2a and b, confirming the entire decomposition of the pristine material into GNPs. The full width at half maximum (FWHM) value of 002 peak increased from 2.2° (pristine MWNT) to 7.6° where the mean inter-planar spacing of 3.60 Å was determined (Fig. 2b). The purified GNPs (purified in alcohol solution: see Methods) evolved a saw-tooth like 002 peak, as shown in Fig. 2c. The mean inter-planar spacing and FWHM value of the unique 002 peak were measured to be 3.48 Å and 4.5°, respectively.

**Figure 3:**
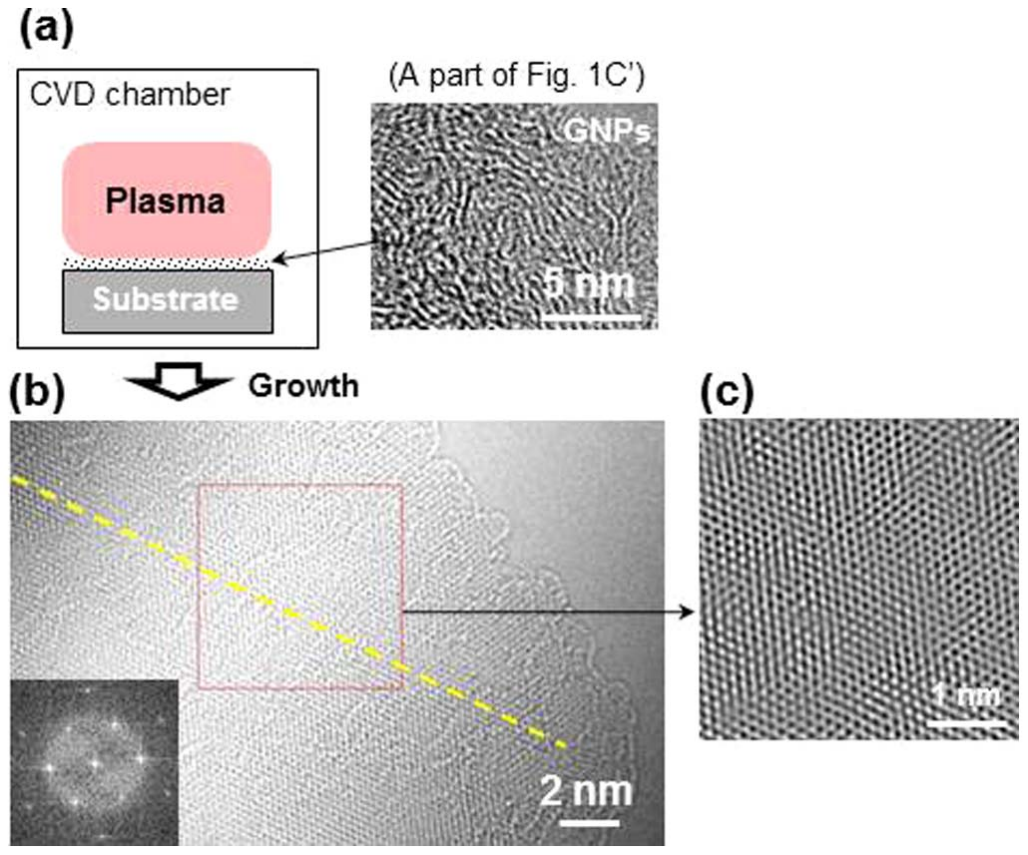
Schematic explaining the seeded growth of graphene and HRTEM images. (a), Schematic explaining the seeded growth of graphene under a plasma condition. (b), Titan HRTEM (operating at 80 kV) image of a graphene sheet. The yellow line passing through the atomic lattices is a visual indication of the single crystal graphene sample. We attribute local mosaic lattices to overlaps of smaller graphene fragments. The inset is FFT diffraction pattern of the region indicated in (b). (c), Inverse FFT image taken from the region indicated in (b).

**Figure 4:**
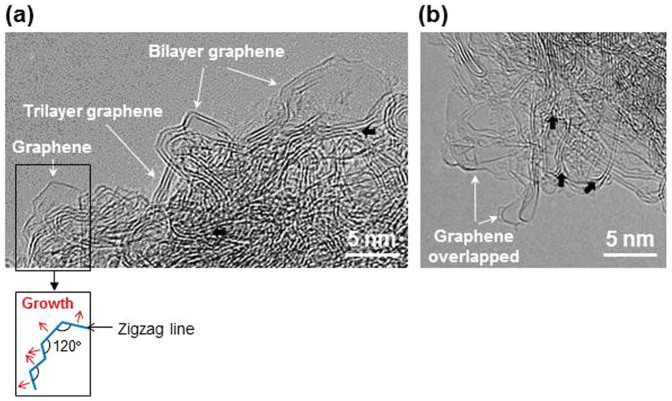
HRTEM images of the graphene samples. Conventional HRTEM (operating at 200 kV) images showing graphene and bi- and trilayer graphene sheets (i.e., thin graphite). The schematic explains the faceted growth of the isolated graphene sheet. Overlapped graphene layers are also observable in (b). Black arrows indicate the intersections of graphene layers.

The GNPs grew to form graphene plates of hundred nm^2^ in area when the nanographene seeds were subject to a direct current plasma, as shown in the HRTEM image in Fig. 3. The characteristic hexagonal atomic lattices (Fig. 3b) and the corresponding unique FFT image yield an hexagonal array of spots (inset) and the edges of monolayer graphene^5^,^8^,^20^ are clearly observable in the image. The prominent edge structures of graphene sheets are also observable in the conventional HRTEM (working at 200 kV) images as shown in Fig. 4a and b where bi- and trilayer graphene sheets (i.e., thin graphite) are also observable. The isolated graphene sheet (Fig. 4a) shows a faceted outline where the crystal edges subtended an angle of 120°. Graphene sheets with lateral dimensions of several tens nm^2^ exhibiting layer edges are evident in Fig. 4b which appear mostly to be curved and overlapped.

**Figure 5:**
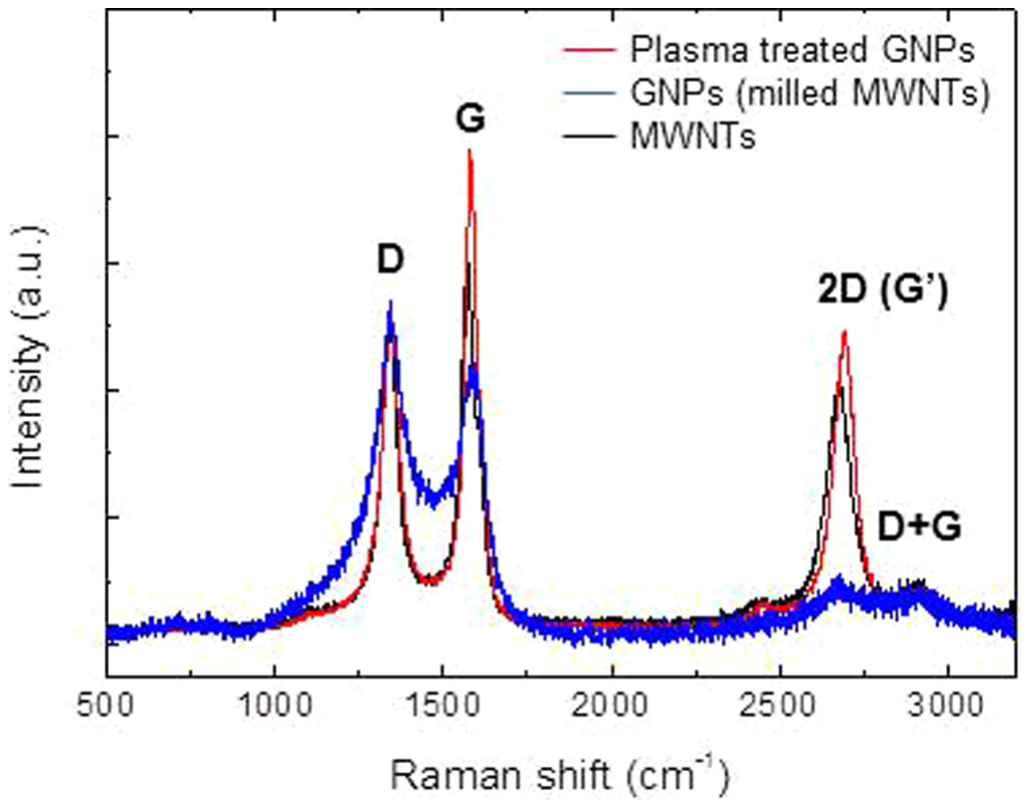
Raman spectra of MWNTs, GNPs, and seeded grown graphene samples. The typical peaks of MWNTs broaden (D and G) or disappear (2D) in the spectrum of GNPs formed by two hours milling. The Raman spectrum of the graphene samples reveals relatively intense narrow peaks.

Figure 5 shows the Raman spectra of MWNTs, GNPs, and plasma treated GNR samples. The typical peaks of MWNTs broaden (D and G) or disappear (2D i.e., G′) in the spectrum of GNPs formed by two hours milling. Intense narrow peaks appear in the Raman spectrum of the graphene samples. The feature is different from those of monolayer graphene^4^,^5^,^21^ as well as graphite^21^ since the relative intensity of the D peak is very strong.

## Discussion

The broad 002 peak with the d-value of 3.60 Å of the milled samples (Fig. 2b) indicates that GNPs exist as randomly oriented (Fig. 2b′) because the inter-planar spacing is larger than those of crystalline graphite, namely, 3.35 Å for AB stacking, 3.44 Å for AA′ stacking and 3.53 Å for AA stacking^1^. The variation of the 002 peak following the purification treatment (Fig. 2b and c) can be explained in terms of stacking between GNPs. The stacking of randomly oriented GNPs (Fig. 2b′) may be due to the surface tension and capillary forces of residual alcohol (Fig. 2b″) or by van der Waals interaction (Fig. 2c′) when completely dry. The packing can be classified as either parallel or random packing according to the distribution of the azimuthal angle between the planes (Fig. 2c′). The former can be regarded as two-dimensionally disordered (turbostratic) structure where the crystallographic relationship^22^ between the graphene layers is absent. The two-dimensionally disordered structure includes locally AB, AA′, and AA (Fig. 1 of ref. 18) stacking and, thus, its mean interplanar spacing lies between 3.35 Å (smallest) for AB stacking and 3.53 Å (largest) for AA stacking^1^. This explains the measured inter-planar spacing of GNPs of ≈3.48 Å. The three-dimensionally disordered structure of the GNPs with a large distribution of the inter-planar spacings, explains the wide base of the 002 peak (Fig. 2c). The explanation for the unique variation of the XRD patterns before and after the solution treatment of GNPs is consistent with the structure of graphene and attraction between the GNPs.

The intersections (black arrows) in Fig. 4a and b of graphene layers indicate that thin graphite may be formed by joining preformed graphene sheets (i.e., independently nucleated) with van der Waals interaction during the CVD growth although we do not exclude the possibility that it was originated from stacked GNP nuclei. We attribute the unique Raman spectrum (strong D peak) of the plasma seeded grown graphene samples to the coexistence of “nano-sized” graphene and thin graphite. The large fraction of the edge structure can increase the relative intensity of the D peak which is related with defects within disordered graphene. The Raman data represent the average signal of numerous graphene and thin graphite nanosheets included within the Raman laser beam where the beam diameter is 1–2 *μ*m (see Methods). The intensity ratio of 2D peak to G peak, I_2D_/I_G_, is about 0.62, and this value is similar to those for the graphene samples grown on Co or Ni (see Table 1 of Ref. 23). The relatively strong G and 2D (G′) peaks of the plasma treated GNP samples compared with those of GNPs as well as MWNTs demonstrates the outgrowth of well-crystallized graphene layers during the secondary growth from GNPs.

We propose a mechanism for the plasma growth of graphene via the lateral growth of the zigzag lines of graphene which subtend angles of 120° (see Fig. 4 of Ref. 24). The zigzag line, which is the closest-packed edge with higher surface energy than that of the armchair edge^24^, can form the template for further growth by the incorporation of carbon species from the plasma. The isolated and faceted graphene grain shown in Fig. 4a resulted from long range lateral growth of the zigzag edges of the graphene crystal.

The overlap of the graphene sheets shown in Fig. 4b could conceivably have occurred during TEM sampling, including a solution treatment where the samples are dispersed in alcohol and deposited on a TEM grid with the conditions of Fig. 2b″ and c′. We attribute the edges shown in Fig. 3b and 4b, unlike the case of the isolated graphene sheet (Fig. 4a), to distortion of the edge lines by stresses. With the evolution of the (002) XRD peak from solution treated GNPs (Fig. 2c), the appearance of overlapping graphene sheets (Fig. 4b) supports our view that it will be difficult to produce monolayer graphene via chemical routes.

The HRTEM images showing the edges of monolayer graphene (Fig. 3b and 4) provide unequivocal evidence of the presence of monolayer graphene. The data are to be compared with the previously reported low magnification TEM images^13^,^17^,^25^,^26^,^27^ where atomic graphene layers cannot be confirmed, cross-sectional HRTEM images where graphene layers appear unclearly^23^,^28^,^29^, and atomic resolution (in-plan) HTRTEM images showing hexagonal lattices^6^,^7^,^8^,^30^,^31^,^32^,^33^,^34^, which are obtainable from bi- or trilayer graphene (i.e., thin graphite)^6^,^8^,^33^. ED patterns showing hexagonal symmetry^13^,^17^,^25^,^27^,^28^,^29^,^30^,^31^,^35^ are also obtainable either from bilayer graphene (Fig. 5b of ref. 6) or single crystal graphite^36^.

In summary, the outgrowth of graphene from the GNP nuclei was achieved at a plasma CVD condition thus producing a new method of forming graphene sheets larger than one hundred nm^2^ in area. Characterization by electron and X-ray diffraction, high-resolution transmission electron microscopy provides direct evidence of the presence of pure monolayer graphene.

## Methods

### Synthesis of graphene

Commercial MWNTs (CM-95, 95 wt% purity, Hanwha nanotech), prepared by VCCVD technique, were used as a starting material for the fabrication of graphene nanopowders (GNPs) to be served as nuclei for the seeded plasma growth of graphene. Diameters and lengths of the MWNT samples were 10–20 nm and 3–20 *μ*m respectively. 1 g of the samples was loaded into a steel jar (Φ 60 × 60 L) with twenty steel balls (each 2 g) and processed by a conventional (Spex) milling apparatus for 1 ~ 6 hours. For the novel CVD growth, the purified GNPs which were prepared on a molybdenum substrate were subjected to a direct current plasma generated at 100 Torr of a 10%CH_4_-90%H_2_ gas mixture for I hour. The temperature of the substrate was kept at 1,200°C.

### Characterization of graphene

The milled samples, i.e., GNPs were confirmed by HRTEM observation as well as X-ray diffraction pattern analysis. The GNPs including metal particles originated from the steel balls were purified through a solution treatment where the samples are dispersed in ethyl alcohol with sonication and the metal impurities are removed by magnets. HRTEM observation for grown graphene sheets were performed by a JEM-2100F operating at 200 kV and a FEI Titan Cubed with aberration corrector and a monochrometer operating at 80 kV. Raman analysis was performed by a Renishaw In-Via Raman Microscope with laser excitation of 532 nm and spot size of 1–2 *μ*m.

## Author Contributions

J.L. designed the experiments and performed data analysis. S.L., Y.P. and K.L. carried out the experiments. Y.K. worked on XRD analysis of the materials. J.K. and B.M. worked on HRTEM observation. J.L. and P.J. co-wrote the paper.

## References

[b1] Lee J.-K. *et al.* The growth of AA graphite on (111) diamond. J. Chem. Phys. 129, 234709 (2008). 19102554 10.1063/1.2975333

[b2] Novoselov K. S. *et al.* Electric field effect in atomically thin carbon films. Science 306, 666–669 (2004). 15499015 10.1126/science.1102896

[b3] Geim A. K. & Novoselov K. S. The rise of graphene. Nat. Mater. 6, 183–191 (2007). 17330084 10.1038/nmat1849

[b4] Kim K. S. *et al.* Large-scale pattern growth of graphene films for stretchable transparent electrodes. Nature 457, 706–710 (2009). 19145232 10.1038/nature07719

[b5] Li X. *et al.* Large-area synthesis of high-quality and uniform graphene films on copper foils. Science 324, 1312–1314 (2009). 19423775 10.1126/science.1171245

[b6] Waldmann D. *et al.* Robust graphene membranes in a silicon carbide frame. ACS Nano 7, 4441–4448 (2013). 23586703 10.1021/nn401037c

[b7] Gass M. H. *et al.* Free-standing graphene at atomic resolution. Nat. Nanotech. 3, 676–681 (2008). 10.1038/nnano.2008.280 18989334

[b8] Cong C., Li K., Zhang X. X. & Yu T. Visualization of arrangements of carbon atoms in graphene layers by Raman mapping and atomic-resolution TEM. Sci. Rep. 3, 1195 (2013). 23378926 10.1038/srep01195 PMC3561624

[b9] Li X., Wang X., Zhang L., Lee S. & Dai H. Chemically derived, ultrasmooth graphene nanoribbon semiconductors. Science 319, 1229–1232 (2008). 18218865 10.1126/science.1150878

[b10] Li D. *et al.* Processable aqueous dispersions of graphene nanosheets. Nat. Nanotech. 3, 101–105 (2008). 10.1038/nnano.2007.451 18654470

[b11] Zhu Y. *et al.* Carbon-based supercapacitors produced by activation of graphene. Science 332, 1537–1541 (2011). 21566159 10.1126/science.1200770

[b12] Eda G., Fanchini G. & Chhowalla M. Large-area ultrathin films of reduced graphene oxide as a transparent and flexible electronic material. Nat. Nanotech. 3, 270–274 (2008). 10.1038/nnano.2008.83 18654522

[b13] Park S. *et al.* Aqueous suspension and characterization of chemically modified graphene sheets. Chem. Mater. 20, 6592–6594 (2008).

[b14] Reynier Y. F., Yazami R. & Fultz B. Thermodynamic of lithium intercalation into graphites and disordered carbons. J. Electrochem. Soc. 151, A422–A426 (2004).

[b15] Thess A. *et al.* Crystalline ropes of metallic carbon nanotubes. Science 273, 483–487 (1996). 8662534 10.1126/science.273.5274.483

[b16] Segal M. Selling graphene by the ton. Nat. Nanotech. 4, 612–614 (2009). 10.1038/nnano.2009.279 19809441

[b17] Choucair M., Thordarson P. & Stride J. A. Gram-scale production of graphene based on solvothermal synthesis and sonication. Nat. Nanotech. 4, 30–33 (2009). 10.1038/nnano.2008.365 19119279

[b18] Lee J.-K. *et al.* Structure of multi-wall carbon nanotubes: AA′ stacked graphene helices. Appl. Phys. Lett. 102, 161911 (2013).

[b19] Iijima S. Growth of carbon nanotubes. Mater. Sci. *&* Eng. B19, 172–180 (1993).

[b20] Girit C. Ö. *et al.* Graphene at the edge: stability and dynamics. Science 323, 1705–1708 (2009). 19325110 10.1126/science.1166999

[b21] Hodkiewicz J. Characterizing Carbon Materials with Raman Spectroscopy, Application note:51901. Thermo Fisher Scientific Inc. (2010).

[b22] Shibuta Y. & Elliott J. A. Interaction between two graphene sheets with a turbostratic orientational relationship. Chem. Phys. Lett. 512, 146–150 (2011).

[b23] Tao L. *et al.* Uniform wafer-scale chemical vapor deposition of graphene on evaporated Cu (111) film with quality comparable to exfoliated monolayer. J. Phys. Chem. C 116, 24068–24074 (2012).

[b24] Lee J.-K. *et al.* Structure of single-wall carbon nanotubes: a graphene helix, accepted in Small. (2014). 10.1002/smll.201400884 24838196

[b25] Yu Q. *et al.* Control and characterization of individual grains and grain boundaries in graphene grown by chemical vapour deposition. Nat. Mater. 10, 443–449 (2011). 21552269 10.1038/nmat3010

[b26] Kosynkin D. V. *et al.* Longitudinal unzipping of carbon nanotubes to form graphene nanoribbons. Nature 458, 872–876 (2009). 19370030 10.1038/nature07872

[b27] Geng D. *et al.* Uniform hexagonal graphene flakes and films grown on liquid copper surface. PNAS 109, 7992–7996 (2012). 22509001 10.1073/pnas.1200339109 PMC3361379

[b28] Sun Z. *et al.* Growth of graphene from solid carbon sources. Nature 468, 549–552 (2010). 21068724 10.1038/nature09579

[b29] Meyer J. C. *et al.* The structure of suspended graphene sheets. Nature 446, 60–63 (2007). 17330039 10.1038/nature05545

[b30] Huang P. Y. *et al.* Grains and grain boundaries in single-layer graphene atomic patchwork quilts. Nature 469, 389–393 (2011). 21209615 10.1038/nature09718

[b31] Kim K. *et al.* Grain boundary mapping in polycrystalline graphene. ACS Nano 5, 2142–2146 (2011). 21280616 10.1021/nn1033423

[b32] Kurasch S. *et al.* Atom-by-atom observation of grain boundary migration in graphene. Nano Lett. 12, 3168–3173 (2012). 22554303 10.1021/nl301141g

[b33] Wang H. *et al.* Unraveling the atomic structure of ultrafine iron clusters. Sci. Rep. 2, 995 (2012). 23251781 10.1038/srep00995 PMC3524523

[b34] Barreiro A. *et al.* Understanding the catalyst-free transformation of amorphous carbon into graphene by current-induced annealing. Sci. Rep. 3, 1115 (2013).

[b35] Liu Z. *et al.* In-plane heterostructures of graphene and hexagonal boron nitride with controlled domain sizes. Nat. Nanotech. 8, 119–124 (2013). 10.1038/nnano.2012.256 23353677

[b36] Ferralis N. *et al.* Low-energy electron diffraction study of potassium adsorbed on single-crystal graphite and highly oriented pyrolytic graphite. Phys. Rev. B 70, 245407 (2004).

